# Incidental findings on 3 T neuroimaging: cross-sectional observations from the population-based Rhineland Study

**DOI:** 10.1007/s00234-021-02852-2

**Published:** 2021-11-29

**Authors:** Valerie Lohner, Ran Lu, Simon J. Enkirch, Tony Stöcker, Elke Hattingen, Monique M. B. Breteler

**Affiliations:** 1 Population Health Sciences, German Center for Neurodegenerative Diseases (DZNE), Venusberg-Campus 1/99, 53127 Bonn, Germany; 2 Clinic for Neuroradiology, University Hospital Bonn, Bonn, Germany; 3 MR Physics, German Center for Neurodegenerative Diseases (DZNE), Bonn, Germany; 4 Department of Physics and Astronomy, University of Bonn, Bonn, Germany; 5 Department of Neuroradiology, University Hospital Frankfurt, Frankfurt, Germany; 6 Institute for Medical Biometry, Informatics and Epidemiology (IMBIE), Faculty of Medicine, University of Bonn, Bonn, Germany

**Keywords:** Population-based study, MRI, Neuroimaging, Observational study, Incidental findings

## Abstract

**Purpose:**

Development of best practices for dealing with incidental findings on neuroimaging requires insight in their frequency and clinical relevance.

**Methods:**

Here, we delineate prevalence estimates with 95% confidence intervals and clinical management of incidental findings, based on the first 3589 participants of the population-based Rhineland Study (age range 30–95 years) who underwent 3 Tesla structural neuroimaging (3D, 0.8 mm^3^ isotropic resolution). Two trained raters independently assessed all scans for abnormalities, with confirmation and adjudication where needed by neuroradiologists. Participants were referred for diagnostic work-up depending on the potential benefit.

**Results:**

Of 3589 participants (mean age 55 ± 14 years, 2072 women), 867 had at least one possible incidental finding (24.2%). Most common were pituitary abnormalities (12.3%), arachnoid cysts (4.1%), developmental venous anomalies (2.5%), non-acute infarcts (1.8%), cavernomas (1.0%), and meningiomas (0.7%). Forty-six participants were informed about their findings, which was hitherto unknown in 40 of them (1.1%). Of these, in 19 participants (48%), a wait-and-see policy was applied and nine (23%) received treatment, while lesions in the remainder were benign, could not be confirmed, or the participant refused to inform us about their clinical diagnosis.

**Conclusion:**

Nearly one-quarter of participants had an incidental finding, but only 5% of those required referral, that mostly remained without direct clinical consequences.

## Introduction

Magnetic resonance imaging (MRI) has been widely used in both research and clinical practice over the past decades. As a consequence, people had to develop best practices for dealing with incidental findings. An incidental finding is a previously unknown abnormality of potential clinical relevance that is unexpectedly discovered and unrelated to the specific research purposes of a study itself [1].

The prevalence of incidental findings on neuroimaging varies across studies depending on the age distribution of participants and the imaging modalities used [2]. So far, population-based studies have reported incidental findings mostly in older people and using 1.5 Tesla neuroimaging [3–7] with only a few studies using at least one 3D imaging sequence [5–7]. To the best of our knowledge, the Study of Health in Pomerania study is the only population-based study that reported on incidental findings on MRI covering a broad age range by including participants aged between 21 and 88 years; however, their imaging protocol was limited to 2D MR images [8].

Based on the large, single-center population-based Rhineland Study, we here report on the prevalence of incidental findings detected on brain neuroimaging using 0.8 mm^3^ isotropic 3D imaging sequences across the adult life span, and provide information about clinical management of incidental findings that were reported back to the participant.

## Methods

### Study population

**Fig. 1:**
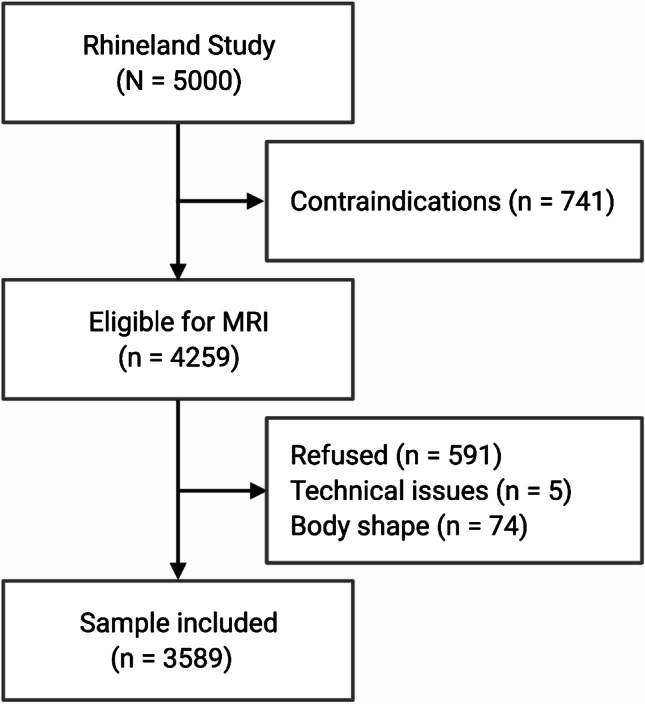
Flowchart showing inclusion and exclusion criteria of the study. *Body shape* indicates participants who did not fit into the MRI scanner

This study is based on all participants who underwent structural brain MRI out of the first 5000 consecutive participants of the Rhineland Study (*n* = 3589, shown in Fig. 1). The Rhineland Study is an ongoing, prospective, single-center, community-based cohort study. All inhabitants aged 30–100 years of two geographically defined areas in Bonn, Germany, are invited to participate in the study. The sole exclusion criterion is insufficient command of the German language to provide informed consent.

### Magnetic resonance imaging data acquisition

MRI data was acquired on 3 Tesla MRI scanners (Siemens Prisma Magnetom, Erlangen, Germany) equipped with an 80 mT/m gradient system and a 64-channel phased-array head-neck coil, including the following in-house developed sequences: a 3D T1-weighted multi-echo magnetization prepared rapid gradient-echo (ME-MPRAGE) sequence (time of acquisition (TA) = 6.5 min, repetition time (TR) = 2560 ms, inversion time (TI) = 1100 ms, flip angle 7°, field of view (FOV) = 256 × 256 mm, 0.8 mm isotropic) [9, 10]; a 3D T2-weighted Turbo-Spin-Echo (TSE) (TA = 4.6 min, TR = 2800 ms, echo time (TE) = 405 ms, FOV = 256 × 256 mm, 0.8 mm isotropic) [11, 12]; and a 3D T2 fluid-attenuated inversion recovery (FLAIR) pulse sequence (TA = 4.5 min, TR = 5000 ms, TE = 393 ms, TI = 1800 ms, FOV = 256 × 256 mm, 1.0 mm isotropic). All sequences employ parallel imaging acceleration with CAIPIRINHA sampling [13] and elliptical sampling [14].

For the initial screening of incidental findings, all images were reconstructed to a resolution of 2.5 mm isotropic to reduce the workload of the reader. When an abnormality was seen, the reader had direct access to the original images for detailed assessment.

### Assessment and clinical management of incidental findings

**Fig. 2:**
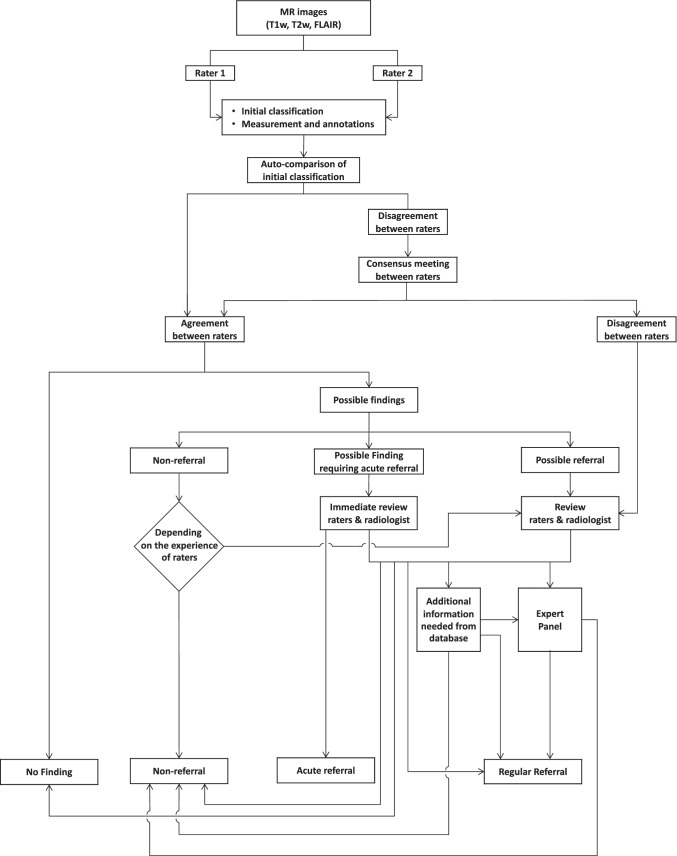
Workflow assessment of incidental findings in the Rhineland Study

The workflow of the assessment of incidental findings in the Rhineland Study is depicted in Fig. 2. Criteria for what constitutes an incidental finding and which findings should be reported back to the participant were developed by an expert committee based on clinical guidelines, state-of-the-art scientific evidence, and ethical considerations (see Table 1). Possible incidental findings that were explicitly, but not exclusively, checked for included infarcts, hemorrhage, malignant tumors, parenchymal brain lesions, intraventricular lesions, pituitary lesions, brainstem lesions, lesions involving a cranial nerve, meningiomas, arachnoid cysts, aneurysms, arteriovenous malformations, cavernous malformations, developmental venous malformations, developmental abnormalities, and white matter hyperintensities that were presumably not due to cerebral small vessel disease (including multiple sclerosis). The latter was based on the dark appearance of white matter hyperintensities on T1-weighted images as well as the clinical experience of the neuroradiologists. Initial readings with this prespecified protocol were performed with OsiriX MD, an image processing application for DICOM images, by two of three independent raters (VL, cognitive neuroscientist with 6 years of experience (until end of study); RL, radiologist with 7 years of experience (until August 2019); specifically trained medical student with 1 year of experience (from August 2019 onwards)). The initial raters had previous experience in MR image reading in clinical routine or for research purposes. Additionally, before the start of the study, they joined the Clinic for Neuroradiology in Bonn for 2 weeks to get more specific training in the detection of brain abnormalities, and had specific training sessions with neuroradiologists (e.g., to distinguish between normal variations and cystic lesions of the pituitary gland). To train new raters, they developed an initial training set including 110 MRI scans from the Rhineland Study, which included both scans with and without abnormalities. The third rater got trained using this initial training set as well as 150 additional random MRI scans from the Rhineland Study. The training set is still increasing in size as raters continue to include interesting cases.

**Table 1:** Protocol for the referral of incidental findings for further diagnostic work-up in the Rhineland Study

Incidental findings that need to be referred for diagnostic work-up
**Acute findings**
Acute infarct
Intracranial hemorrhage
**Mass**
Malignant tumors, including glioma
Any brain parenchymal lesion (including cystic) with oedema/hydrocephalus/midline shift/nerve or vessel impairment
Any intraventricular lesion that might cause a hydrocephalus
Solid/semi-solid pituitary lesion > 1 cm or any cystic lesion with mass effect > 1 cm
Solid/semi-solid lesion or any cystic lesion with mass effect in brainstem
Lesions with involvement of a cranial nerve
Meningiomas
Convexity meningiomas > 2 cm
All non-convexity meningiomas regardless of size
**Vascular disease**
Aneurysm with PHASES score ≥ 5
Aneurysm in posterior circulation including posterior communicating artery with PHASES score < 5 should be discussed in the Panel to make the final decision
Sub-acute intracranial hemorrhage bleeding (including subdural hematoma, epidural hematoma, intracerebral hemorrhage, subarachnoid hemorrhage, intraventricular hemorrhage)
**Incidental findings that do not need diagnostic work-up and are not communicated to the participant**
Mass and vascular diseases not mentioned in the list above
Arachnoid cysts
Non-acute cerebral infarcts
Arteriovenous malformations
Cavernous malformations
White matter hyperintensities, including multiple sclerosis
Developmental abnormalities

The initial ratings were done blinded to the medical history of participants, usually within 1 working day by at least one of the raters. Next, both ratings were compared. In case of persistent disagreement, an incidental finding that possibly would require referral, or whenever further clarification was needed, an experienced (neuro-)radiologist also read the images and made a final decision on the classification of the finding (SJE, radiologist with 7 years of experience; EH, neuroradiologist with 23 years of experience). All judgements were solely made on the basis of the MRI scans.

The decision whether or not to refer a participant with an incidental finding to a medical specialist for clinical work-up depended on the potential benefit for the participant, which was defined a priori by the expert committee mentioned above (Table 1). In case of ethically challenging findings, further experts could be consulted. When referral was needed, a study physician informed the participant and, with the consent of the participant, their general practitioner. Note that we only received feedback on the detected brain abnormality from the persons who we approached for referral. Therefore, we cannot exclude that some of the non-referred lesions were already known to the participant, and therefore in sensu stricto not an incidental finding, even though they had not been reported during the interview.

To obtain information on clinical management of referred abnormalities, we asked the participants to send relevant medical letters or to give consent for us to contact their practitioner to review medical records directly. We only considered clinical diagnoses made by medical specialists after clinical neuroimaging.

### Assessment of demographic variables

Hypertension was defined as systolic blood pressure ≥ 140 mmHg, diastolic blood pressure ≥ 90 mmHg, or antihypertensive medication use; diabetes as fasting plasma glucose level ≥ 7 mmol/l, HbA1_c_ ≥ 6.5%, or use of antidiabetic medication. History of multiple sclerosis and stroke, smoking status (current/non-smoker), and education (low, ISCED 0–3; middle, ISCED 4–6; high, ISCED 7–8)[15] was self-reported.

### Data availability

The data for this manuscript are not publicly available due to data protection regulations. Access to data can be provided to scientists in accordance with the Rhineland Study’s Data Use and Access Policy. Requests for additional information and/or access to the datasets can be send to RS-DUAC@dnze.de.

### Statistical analysis

We calculated the prevalence with 95% confidence intervals (CI) for each incidental finding in our study population. For the most frequent incidental findings, we further evaluated whether prevalence differed between sexes and across age using logistic regression. Multiple similar incidental findings within one participant were counted as a single finding (e.g., multiple arachnoid cysts). *P*-values < 0.05 were considered as statistically significant. All statistical analyses were performed using R version 4.0.2 [16].

## Results

Mean age of the study population was 55 ± 14 years, 58% were women (Table 2). Men compared to women were on average more often higher educated (65 vs. 48%, *p* = 0.001), were more likely to have diabetes (6 vs. 3%, *p* < 0.001) and hypertension (40 vs. 34%, *p* < 0.001), and a higher body mass index (26.1 vs. 25.3, *p* < 0.001). Participants who underwent MRI were on average younger (55 vs. 56 years, *p* < 0.001), more often higher educated (55 vs. 47%, *p* < 0.001), were less likely to have diabetes (5 vs. 7%, *p* < 0.001), or hypertension (37 vs. 42%, *p* < 0.001), and had a lower body mass index (25.6 vs. 26.7, *p* < 0.001), compared to those who did not. Also, more men than women (47 vs. 42%, *p* = 0.005) were excluded from or refused MRI.

**Table 2:** Characteristics of the study population

Characteristics		Whole cohort	Sample with MRI				Sample without MRI	
			Overall	Women	Men	*p*-value†		*p*-value‡
		*n* = 5000	*n* = 3589	*n* = 2072	*n* = 1517		*n* = 1517	
Age in years, mean ± SD		55 ± 14	54 ± 14	55 ± 14	54 ± 14	0.23	56 ± 15	< 0.001
Women, *n* (%)		2824 (56)	2072 (58)				752 (53)	< 0.01
Age group, *n* (%)						0.11		< 0.001
	30–39 years	833 (17)	627 (17)	335 (16)	292 (19)		206 (15)	
	40–49 years	926 (19)	676 (19)	390 (19)	286 (19)		250 (18)	
	50–59 years	1358 (27)	988 (28)	593 (29)	395 (26)		370 (26)	
	60–69 years	1009 (20)	736 (21)	440 (21)	296 (20)		273 (19)	
	70–79 years	666 (13)	450 (13)	252 (12)	198 (13)		216 (15)	
	80 + years	208 (4)	112 (3)	62 (3)	50 (3)		96 (7)	
Education, *n* (%)						< 0.01		< 0.001
	Low	101 (2)	61 (2)	48 (2)	13 (1)		40 (3)	
	Middle	2232 (45)	1532 (43)	1015 (49)	517 (34)		700 (50)	
	High	2621 (53)	1969 (55)	988 (48)	981 (65)		652 (47)	
Diabetes, *n* (%)		261 (5)	161 (5)	67 (3)	94 (6)	< 0.001	100 (7)	< 0.001
Hypertension, *n* (%)		1867 (38)	1283 (37)	684 (34)	599 (40)	< 0.001	584 (42)	< 0.001
Smoking, *n* (%)		621 (12)	459 (13)	252 (12)	207 (14)	0.22	162 (12)	0.09
BMI (kg/m^2^), mean ± SD		25.9 ± 4.5	25.6 ± 4.2	25.3 ± 4.7	26.1 ± 3.5	< 0.001	26.7 ± 5.2	< 0.001
Self-reported MS, *n* (%)		25 (1)	21 (1)	15 (1)	6 (0)	0.19	4 (0)	0.13
Self-reported stroke, *n* (%)		78 (2)	47 (1)	24 (1)	23 (2)	0.33	31 (2)	0.10

*SD*, standard deviation; *BMI*, body mass index; *MS*, multiple sclerosis

^†^*P*-values are adjusted for age where applicable and show differences between women and men

^‡^*P*-values are adjusted for age and sex where applicable and show differences between participants with and without MRI

In total, 867 of 3589 participants had at least one possible incidental finding (24.2% [95% CI 22.8–25.6%]) (Table 3). This did not differ between women (505 of 2072 with an incidental finding (24.4% [95% CI 22.5–26.3%])) and men (362 of 1517 (23.9% [95% CI 21.7–26.1%])) (*p* = 0.764). The maximum number of incidental findings for a single person was four; one participant had an arachnoid cyst, a developmental venous anomaly, a cavernoma, and a possibly malignant lesion; another participant had an arachnoid cyst, cystic lesion of the pituitary gland, inflammatory WM lesions, and cystic lesions around the brainstem. Most frequent incidental findings were pituitary abnormalities (12.3% [95% CI 11.3–13.5%]), arachnoid cysts (4.1% [95% CI 3.5–4.8%]), developmental venous anomalies (2.5% [95% CI 2.0–3.0%]), non-acute infarcts (1.8% [95% CI 1.4–2.3%]), cavernomas (1.0% [95% CI 0.7–1.4%]), and meningiomas (0.7% [95% CI 0.5–1.1%], mean size of the largest dimension, 14.9 ± 6.8 mm). Men had more non-acute infarcts, more arachnoid cysts, and more developmental abnormalities than women (2.3 vs. 1.4%, *p* = 0.040; 5.2 vs. 3.3%, *p* = 0.006; 0.7 vs. 0.1%, *p* = 0.015, respectively). Women had slightly more meningiomas, but because of small numbers, the difference was only borderline significant (1.0 vs. 0.4%, *p* = 0.056). The presence of non-acute infarcts increased with age (prevalence odds ratio (OR) 1.06 [95% CI 1.04–1.08] per year, *p* < 0.001), as did the frequency of cavernomas (OR 1.03 [95% CI 1.00–1.05] per year, *p* = 0.044), and of meningiomas (OR 1.05 [95% CI 1.02–1.08] per year, *p* = 0.002). For other incidental findings, we saw no effect of age on prevalence.

**Table 3:** Overview of incidental findings in the Rhineland Study

Incidental finding	Overall (n=3589)	Women (n=2072)	Men (n=1517)	p-value†
Any, n (%)	867 (24.2)	505 (24.2)	362 (23.9)	0.08
Pituitary abnormality, n (%)	443 (12.3)	267 (12.9)	176 (11.6)	0.25
Arachnoid cyst ¥, n (%)	148 (4.1)	69 (3.3)	79 (5.2)	0.01
Developmental venous abnormality ¶, n (%)	89 (2.5)	50 (2.4)	39 (2.6)	0.77
Non-acute infarcts #, n (%)	64 (1.8)	29 (1.4)	35 (2.3)	0.04
Other þ, n (%)	43 (1.2)	23 (1.1)	20 (1.3)	0.55
Cavernoma ‡, n (%)	35 (1.0)	22 (1.1)	13 (0.9)	0.56
Other mass, n (%)	30 (0.8)	20 (1.0)	10 (0.7)	0.32
Meningioma §, n (%)	26 (0.7)	20 (1.0)	6 (0.4)	0.06
Hemorrhage *, n (%)	14 (0.4)	7 (0.3)	7 (0.5)	0.55
Developmental abnormality, n (%)	14 (0.4)	3 (0.1)	11 (0.7)	0.02
MS-like lesions, n (%)	16 (0.4)	9 (0.4)	7 (0.5)	0.97
Unknown white matter disease, n (%)	11 (0.3)	8 (0.4)	3 (0.2)	0.33
Aneurysm ¬, n (%)	8 (0.2)	4 (0.2)	4 (0.3)	0.66
Other vascular disease, n (%)	7 (0.2)	4 (0.2)	3 (0.2)	0.97
Inflammatory white matter disease, n (%)	6 (0.2)	4 (0.2)	2 (0.1)	0.67
Malignant lesion, n (%)	3 (0.1)	1 (0.0)	2 (0.1)	0.41
Arteriovenous malformation, n (%)	2 (0.1)	2 (0.1)	0 (0.0)	0.99
Intraventricular lesion, n (%)	2 (0.1)	1 (0.0)	1 (0.1)	0.82
Brainstem lesion, n (%)	4 (0.1)	2 (0.1)	2 (0.1)	0.77
Cranial nerve lesion, n (%)	3 (0.1)	1 (0.0)	2 (0.1)	0.44

Other also includes post-operative changes (n = 19) and post-traumatic defects (n = 6)

¥ There were 161 arachnoid cysts in 148 participants, 137 participants had one arachnoid cyst, 9 had two, and one had three

¶ There were 92 developmental venous abnormalities (DVA) in 89 participants, 86 had one DVA, three had two DVAs

# There were 84 non-acute infarcts in 64 participants, 49 participants had one post-ischemic lesion, twelve had two, two had three, and one had five non-acute infarcts

þ There were 44 other abnormalities in 43 participants, 40 had one abnormality, one had two

‡ There were 40 cavernomas in 35 participants, 33 had one cavernoma, one had two cavernomas, and one had five

§ There were 27 meningioma in 26 participants, 25 had one meningioma, one had two

* There were 28 hemorrhages in 14 participants, eight had one hemorrhage, one had two, two had three, and three had four hemorrhages

¬ There were nine aneurysms in eight participants. Seven had one aneurysm, one had two aneurysms

† P-values are adjusted for age and show differences between women and men

Most of the 433 pituitary anomalies that we found were pituitary cysts (mostly pars intermedia cysts; 95.9% [95% CI 93.7–97.6%]); the remainder were (semi-)solid lesion with or without a mass effect, most likely to be microadenomas. The prevalence of pituitary cysts did not significantly differ between men (10.9% [95% CI 9.4–12.6%]) and women (12.5% [95% CI 11.1–14.0%]) (*p* = 0.174) and was stable across the adult life span (OR 1.00 [95% CI 0.99–1.01] per year, *p* = 0.805). The prevalence of other pituitary anomalies did not differ between sexes (women 0.4% [95% CI 0.2–0.8%]; men 0.7% [95% CI 0.3–1.2%]; *p* = 0.187) but increased with age (OR 1.04 [95% CI 1.01–1.08] per year, *p* = 0.024).

The raters had initial disagreement in the reading of the MR images in approximately 12% of the cases, where one of the raters had missed an abnormality. Persistent disagreement occurred in less than 1%, where clarification by the neuroradiologist was needed.

### Referrals and clinical management

Table 4 shows the subsequent clinical management of the 40 participants who we referred for further diagnostic work-up. They underwent clinical MRI which led to a wait-and-see policy for 19, and treatment for nine participants. In four participants, the findings were confirmed but classified as benign lesions that did not require further therapy or follow-up. Three participants refused to give information on their clinical diagnosis.

**Table 4:** Clinical management of 42 different incidental findings that were reported back to 40 participants

Incidental findings and type of management	Clinical diagnosis	Number of findings
**Meningioma** †		**9**
Wait and see	Meningioma	7
Surgery	Meningioma	2
**Brainstem lesion**		**6**
Wait and see	Atypical cystic lesion (*n* = 1), unclear lesion (*n* = 1), calcified cavernous malformation or microbleeding (*n* = 1)	3
No therapy needed	Vascular encephalopathy (*n* = 1), cavernous malformation (*n* = 1)	2
Not confirmed	/	1
**Aneurysm** ‡		**7**
Operative clipping	Aneurysm	4
Endovascular coiling	Aneurysm	1
Not confirmed	/	1
Refused to give information	Unknown	1
**Other mass**		**5**
Wait and see	Cystic porencephalic lesion (*n* = 1), unclear lesion (*n* = 1)	2
Surgery	Pilocytic astrocytoma	1
No therapy needed	Benign cyst aqueduct	1
Refused clinical follow-up	Unknown	1
**Pituitary abnormalities**		**3**
Wait and see	Macroadenoma	3
**Cranial nerve lesion**		**2**
Wait and see	Vestibular schwannoma (*n* = 1), cystic lesion (*n* = 1)	2
**Intraventricular mass**		**2**
Not confirmed	/	1
Refused to give information	Unknown	1
**Possible malignant lesion**		**3**
Wait and see	Unclear lesion	2
No therapy needed	Gliosis	1
**Unclear lesions**		**3**
Not confirmed	/	2
Wait and see	Unclear lesion	1
**Venous malformation**		**1**
Wait and see	Hemangioma	1
**Dural fistula**		**1**
Surgery	Dural fistula	1

Multiple similar incidental findings within one participant were counted as single finding (e.g., aneurysms)

^†^Eight participants had non-convexity meningiomas, one had a convexity meningioma bigger than 2 cm in the longest dimension

^‡^Based on the PHASES score (mean PHASES score 6.6, SD = 1.4). In one of these participants, image quality was insufficient and there was an artefact around the suspicious aneurysm

The initial finding on basis of the research examination was not confirmed in five of the 40 participants (13% [95% CI 4–27%]). In these five participants, we found signal changes of unclear pathogenesis. In two participants, we found cystic lesions of which one could possibly affect the brainstem and the other might possibly cause a hydrocephalus. In two participants, we observed signal changes around the amygdala and in another one changes in the anterior communicating artery which were surrounded by an artefact. In all those cases, we could not rule out malignant pathology and therefore referred these participants for clinical work-up.

Additionally, we found abnormalities that would have required referral according to our protocol in six participants, but were already known and under treatment, and hence by definition no incidental finding.

We did not find any acute lesions that required immediate medical attention, nor any ethically challenging findings for which we would have needed to consult further experts.

## Discussion

In this population-based neuroimaging study among 3589 participants of the Rhineland Study, we found incidental brain abnormalities on MRI in approximately one-quarter of all participants, with pituitary cysts being most common. Based on a prespecified protocol, we had to refer 1.1% of all participants for further diagnostic work-up, mostly because of meningiomas, lesions affecting the brainstem, aneurysms, and mass. Subsequent clinical management in the majority of these participants was confined to a wait-and-see policy. One-fifth of those who were referred, or 0.3% of the total sample that had brain imaging, underwent treatment which was successful and without complications.

Consistent with previous reports [5, 17–19], we found that men had more arachnoid cysts and non-acute infarcts than women, whereas women had slightly more meningiomas, and that the prevalence of non-acute infarcts and meningiomas increased with age. Contrary to a previous population-based study in older adults, we observed an effect of age on the prevalence of cavernomas [20]. However, the other study only assessed axial T2*-weighted (slice thickness 3.3 mm) or standard T2-weighted images, and their reported prevalence of 0.4% may have been too low to detect age-dependencies.

The prevalence of incidental findings is highly dependent on imaging modalities, with more abnormalities being detected when using at least one high spatial resolution 3D sequence [2, 21]. We found pituitary cysts in 11.8% and arachnoid cysts in 4.1% of our population, which is indeed much higher compared to previous studies reporting frequencies in the range of 0.8–1.8% and 1.4–3.6%, respectively [4–8]. This is likely due to the high spatial resolution of our 3D T2-weighted sequence. The prevalence of aneurysms (0.2%) in our cohort is low compared to previous large cohort studies [4–6], which, however, used different imaging modalities, including 2D T2-weighted images or time-of-flight angiography. Our imaging protocol was indeed not optimized to detect aneurysms. Particularly, our highly accelerated 3D T2-weighted sequence is prone to pulsation artefacts interfering with regular intraluminal flow void, making it less suitable for detecting aneurysms.

Discrepancies in prevalence estimates of incidental findings might also be due to classification of what constitutes an incidental finding. For example, we did not include lacunar stroke in non-acute infarcts nor did we track any normal variants (e.g., megacisterna magna).

While the raters initially disagreed in approximately 12% of the cases, this persisted in only less than 1% after an initial consensus meeting. This highlights that it is common for non-radiologist raters to miss small abnormalities on brain MRI scans, and the importance of the four-eye-principle in the reading of MR images in large cohort studies. As the clinical neuroradiologists were not involved in the initial ratings, we could not compare their performance with that of the study raters.

Following our protocol to only refer participants for clinical work-up if this would be of clear potential benefit for the person involved, we only referred 1.1% of the participants suggesting that most abnormalities have no direct clinical consequence. This is in line with reports on potentially clinically relevant incidental findings in adults from a recent meta-analysis and another German cohort [8, 21]. Five of the findings we referred were not confirmed on clinical MRI. Here, we could not rule out possible malignant pathogenesis based on our MRI sequences which were developed for the specific research purposes of the Rhineland Study and not used in clinical settings before. Our prospective follow-up may show whether the lesions we found were indeed false-positive ratings, or that our sequences are more sensitive to subtle changes that are not detectable yet on clinical MRI scans.

Participants included in this study were relatively healthy, as we had to exclude older and sicker people due to MRI contraindications. Additionally, roughly 14% of eligible participants refused MRI. This may have resulted in a further selection bias and the prevalence estimates should be considered a conservative estimate of the true population prevalence of incidental abnormalities.

Major strengths of this study were that it involves a large number of participants drawn from a population-based cohort with a wide age range. We performed state-of-the-art brain MRI including 3D T2-weighted, 3D T1-weighted, and 3D FLAIR sequences, contacted those affected with abnormalities, and followed up concerning their clinical course. All images were reviewed within one working day by at least one experienced reader.

When interpreting the results of this study, some issues should be considered. Our rating of incidental findings was limited to T1-weighted, T2-weighted, and FLAIR images and we did not apply any contrast agents. This may have restricted the number of incidental findings detected and may explain some differences in our prevalence estimates compared to previous studies. Furthermore, we do not have longitudinal data on the natural course of incidental findings yet. The prospective nature of the Rhineland Study, however, will allow us to obtain these in the future.

## Conclusions

In conclusion, incidental findings on neuroimaging across the adult life span are common, yet direct clinical consequences are rare. With the number of research studies using high spatial resolution 3D MR neuroimaging sequences rapidly increasing, it is important to have prespecified guidelines on assessing and managing incidental findings. Our procedure and findings can help guiding in the further development of protocols for new research studies.

## Data Availability

The data for this manuscript are not publicly available due to data protection regulations. Access to data can be provided to scientists in accordance with the Rhineland Study’s Data Use and Access Policy. Requests for additional information and/or access to the datasets can be send to RS-DUAC@dzne.de.

## References

[1] Wolf SM, Lawrenz FP, Nelson CA, Kahn JP, Cho MK, Clayton EW, Fletcher JG, Georgieff MK, Hammerschmidt D, Hudson K, Illes J, Kapur V, Keane MA, Koenig BA, Leroy BS, McFarland EG, Paradise J, Parker LS, Terry SF, Van Ness B, Wilfond BS (2008) Managing incidental findings in human subjects research: analysis and recommendations. J Law Med Ethics 36(2):219–48, 11. 10.1111/j.1748-720X.2008.00266.x 10.1111/j.1748-720X.2008.00266.x PMC2575242 18547191

[2] Orme NM, Fletcher JG, Siddiki HA, Harmsen WS, O’Byrne MM, Port JD, Tremaine WJ, Pitot HC, McFarland EG, Robinson ME, Koenig BA, King BF, Wolf SM (2010). Incidental findings in imaging research: evaluating incidence, benefit, and burden. Arch Intern Med.

[3] Vernooij MW, Ikram MA, Tanghe HL, Vincent AJ, Hofman A, Krestin GP, Niessen WJ, Breteler MM, van der Lugt A (2007). Incidental findings on brain MRI in the general population. N Engl J Med.

[4] Sandeman EM, Hernandez Mdel C, Morris Z, Bastin ME, Murray C, Gow AJ, Corley J, Henderson R, Deary IJ, Starr JM, Wardlaw JM (2013). Incidental findings on brain MR imaging in older community-dwelling subjects are common but serious medical consequences are rare: a cohort study. PLoS ONE.

[5] Bos D, Poels MM, Adams HH, Akoudad S, Cremers LG, Zonneveld HI, Hoogendam YY, Verhaaren BF, Verlinden VJ, Verbruggen JG, Peymani A, Hofman A, Krestin GP, Vincent AJ, Feelders RA, Koudstaal PJ, van der Lugt A, Ikram MA, Vernooij MW (2016). Prevalence, clinical management, and natural course of incidental findings on brain MR images: the population-based Rotterdam Scan Study. Radiology.

[6] Haberg AK, Hammer TA, Kvistad KA, Rydland J, Muller TB, Eikenes L, Garseth M, Stovner LJ (2016). Incidental intracranial findings and their clinical impact; the HUNT MRI study in a general population of 1006 participants between 50–66 years. PLoS ONE.

[7] Boutet C, Vassal F, Celle S, Schneider FC, Barthelemy JC, Laurent B, Barral FG, Roche F (2017). Incidental findings on brain magnetic resonance imaging in the elderly:the PROOF study. Brain Imaging Behav.

[8] Hegenscheid K, Seipel R, Schmidt CO, Volzke H, Kuhn JP, Biffar R, Kroemer HK, Hosten N, Puls R (2013). Potentially relevant incidental findings on research whole-body MRI in the general adult population: frequencies and management. Eur Radiol.

[9] van der Kouwe AJW, Benner T, Salat DH, Fischl B (2008). Brain morphometry with multiecho MPRAGE. Neuroimage.

[10] Brenner D, Stirnberg R, Pracht ED, Stocker T (2014). Two-dimensional accelerated MP-RAGE imaging with flexible linear reordering. MAGMA.

[11] Busse RF, Brau AC, Vu A, Michelich CR, Bayram E, Kijowski R, Reeder SB, Rowley HA (2008). Effects of refocusing flip angle modulation and view ordering in 3D fast spin echo. Magn Reson Med.

[12] Mugler JP (2014). Optimized three-dimensional fast-spin-echo MRI. J Magnet Resonan Imag : JMRI.

[13] Breuer FA, Blaimer M, Mueller MF, Seiberlich N, Heidemann RM, Griswold MA, Jakob PM (2006). Controlled aliasing in volumetric parallel imaging (2D CAIPIRINHA). Magn Reson Med.

[14] Bernstein MA, Fain SB, Riederer SJ (2001). Effect of windowing and zero-filled reconstruction of MRI data on spatial resolution and acquisition strategy. J Magnet Resonan Imag : JMRI.

[15] UNESCO (2012) Institute for Statistics: International Standard Classification of Education ISCED 2011. Montréal

[16] R Core Team (2020) R: a language and environment for statistical computing. In: R Foundation for Statistical Computing, editor. Vienna, Austria

[17] Reeves MJ, Bushnell CD, Howard G, Gargano JW, Duncan PW, Lynch G, Khatiwoda A, Lisabeth L (2008). Sex differences in stroke: epidemiology, clinical presentation, medical care, and outcomes. Lancet Neurol.

[18] Wiemels J, Wrensch M, Claus EB (2010). Epidemiology and etiology of meningioma. J Neurooncol.

[19] Oya S, Kim SH, Sade B, Lee JH (2011). The natural history of intracranial meningiomas. J Neurosurg.

[20] Flemming KD, Graff-Radford J, Aakre J, Kantarci K, Lanzino G, Brown RD, Mielke MM, Roberts RO, Kremers W, Knopman DS, Petersen RC, Jack CR (2017). Population-based prevalence of cerebral cavernous malformations in older adults: Mayo Clinic Study of Aging. JAMA Neurol.

[21] Gibson LM, Paul L, Chappell FM, Macleod M, Whiteley WN, Al-Shahi Salman R, Wardlaw JM, Sudlow CLM (2018). Potentially serious incidental findings on brain and body magnetic resonance imaging of apparently asymptomatic adults: systematic review and meta-analysis. BMJ.

